# Role of microRNA and Oxidative Stress in Influenza A Virus Pathogenesis

**DOI:** 10.3390/ijms21238962

**Published:** 2020-11-25

**Authors:** Md Mamunul Haque, Dhiraj P. Murale, Jun-Seok Lee

**Affiliations:** 1 Molecular Recognition Research Center, Korea Institute of Science and Technology (KIST), Seoul 02792, Korea; mamun@chembiol.re.kr (M.M.H.); dhiraj.murale@chembiol.re.kr (D.P.M.); 2 Bio-Med Division, KIST-School UST, Seoul 02792, Korea

**Keywords:** microRNA, oxidative stress, influenza A virus

## Abstract

MicroRNAs (miRNAs) are non-coding RNAs that regulate diverse cellular pathways by controlling gene expression. Increasing evidence has revealed their critical involvement in influenza A virus (IAV) pathogenesis. Host–IAV interactions induce different levels of oxidative stress (OS) by disrupting the balance between reactive oxygen species (ROS) and antioxidant factors. It is thought that miRNA may regulate the expression of ROS; conversely, ROS can induce or suppress miRNA expression during IAV infection. Thus, miRNA and OS are the two key factors of IAV infection and pathogenesis. Accordingly, interactions between OS and miRNA during IAV infection might be a critical area for further research. In this review, we discuss the crosstalk between miRNAs and OS during IAV infection. Additionally, we highlight the potential of miRNAs as diagnostic markers and therapeutic targets for IAV infections. This knowledge will help us to study host–virus interactions with novel intervention strategies.

## 1. Introduction

More than 85% of the human genome is transcribed into RNA transcripts, whereas just approximately <3% encode proteins [1]. A large proportion of the RNA transcripts of the human genome have no protein coding capacity (non-coding RNAs, ncRNAs) in terms of acting on multiple cellular processes. MicroRNA (miRNA or miR; around 22 nucleotide long) is one of the major ncRNAs of the human genome that regulates various cellular processes like cell proliferation, viral pathogenesis, and apoptosis. The miRNAs regulate these processes by degrading target mRNAs or inhibiting translation [2]. A growing body of evidence demonstrates that the biogenesis and expression of miRNA is altered in response to reactive oxygen species (ROS). Under biological conditions, cells produce modest amounts of ROS when foreign intruders are detected by means of the anti-oxidation pathway. Disproportions among these ROS and anti-oxidation pathways cause the excessive production of ROS, which is referred to as oxidative stress (OS). In OS conditions, cells produce many ROS, including free radicals and non-radicals species (mainly superoxide anions). It has been reported that superoxides are generated by the nonstructural protein (NS1) of avian influenza (AI) viruses, which indicates a link between ROS levels and influenza virus infection [3]. Influenza viruses have four distinct classes: A, B, C, and D. Among them, the influenza A viruses (IAVs) infect diverse host species and represent greater threat compared to the other types. The natural reservoirs of IAVs are wild aquatic birds, but they also commonly infect humans, pigs, canines, bats, aquatic mammals, etc. IAVs have already caused several pandemics, which have resulted in seasonal epidemics and economic losses annually around the world.

IAV pathogenesis constitutes the binding and entering of viruses into host cells, overcoming antiviral responses, replicating, and translating the viral genome into the host cells, and finally releasing the virion to transmit between individuals. All these steps are determined by interactions between the virus and host cell factors, including miRNA and OS. These host factors are diverse in their activation or expression levels. This review summarizes the detailed information about the roles of miRNAs and ROS in IAV infection. Moreover, the potential of miRNAs as diagnostic markers and therapeutic targets for IAV infection is also discussed.

## 2. Functional Involvement of miRNAs in Host–Virus Interactions

High-throughput studies have discovered several miRNA expression patterns with different IAV infection strains in various cellular models. The miRNA regulates a broad range of host–IAV interactions, including immune responses, cell cycle regulation, apoptosis, etc. These interactions also regulate the replication, translation, and pathogenesis of the viruses. All of the above processes by miRNA are temporal, viral strain-specific, and host-dependent.

### 2.1. miRNAs Regulate Cellular Pathways upon IAV Infection

**Figure 1:**
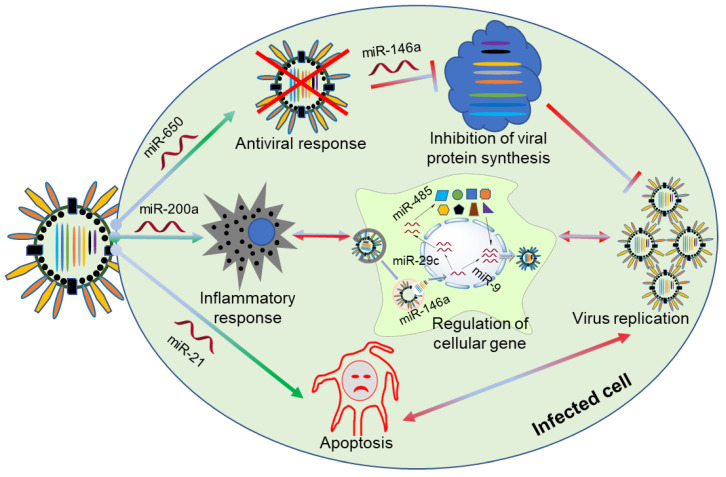
The involvement of miRNAs that modulate host-mediated pathways. IAV infection alters the expression profiles of certain cellular miRNAs related to viral replication, pathogenesis, and antiviral responses. Certain miRNAs have important roles in inflammatory responses and apoptotic pathways as well.

The expression of host miRNA by viral infection influences host immune responses through both transcriptional and translational mechanisms. Several studies have already profiled some of these significantly altered miRNA expression patterns during IAV infection in infected cells [4,5,6,7]. These can be attributed to the host immune signaling, inflammatory functions, cell differentiation, cell cycle, apoptosis, and antiviral defenses (Figure 1). For example, IAV infection reduces the expression of miR-4276 and miR-200a to induce apoptosis [8] and damage inflammatory responses [9], respectively. Many miRNAs are involved in antiviral responses [10] by targeting host or viral genes (Table 1).

Cellular miRNAs are induced or suppressed by viral infection to regulate host responses. The innate immune response is the first line of defense against viruses. Accordingly, some miRNAs are altered to modulate IAV pathogenesis. For instance, miR-650 is decreased upon IAV infection to support antiviral responses [11]. In addition, some miRNAs play critical roles in regulating pro-inflammatory signaling pathways during IAV pathogenesis. For example, miR-29c [12] and miR-451 [13] are increased upon IAV infection to downregulate the inflammatory responses of cells. Here, Table 1 summarizes the critical miRNAs in IAV–host interactions according to their regulatory functions and target genes in the host.

### 2.2. miRNAs Effect on Viral Pathogenesis

**Figure 2:**
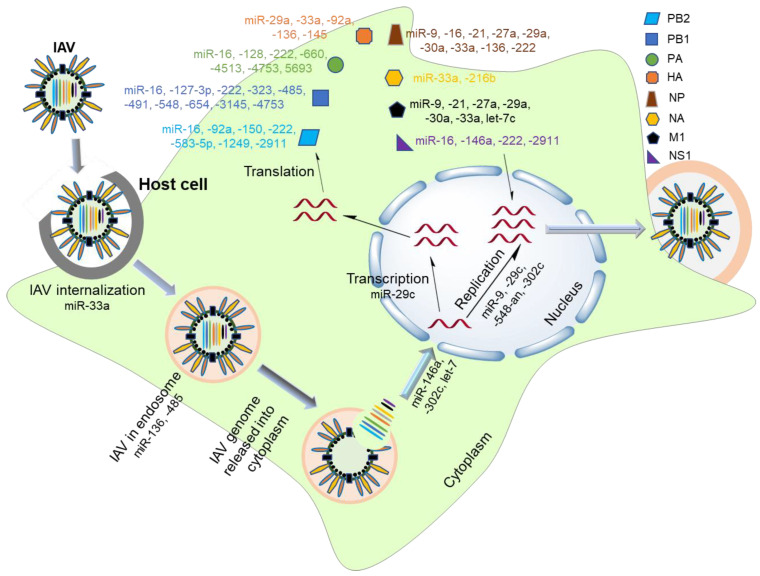
Involvement of miRNAs in the pathogenesis of an influenza virus. The IAV invades the cell and is taken up by the endosome. Upon the rupture of the endosome, the virus releases its genetic material into the nucleus. In turn, transcription, translation, and replication processes are carried out, followed by the release of newly synthesized viral progenies. Several microRNAs regulate these pathways to inhibit or promote IAV pathogenesis.

From the perspective of IAV infection, cellular miRNA expression is critically important for virus–host interactions like viral entry, replication, translation, and transmission. Many of the miRNAs target and modulate the expression of viral genes in IAV pathogenesis. Some microRNAs have a unique target on polymerase complex genes such as miR-150, -583-5p and -1249 for PB2, miR-127-3p, -323, -491, and -654 for polymerase basic protein 1 (PB1), miR-128, -660, 4513 and 5693 for polymerase acidic protein (PA) [4,10,20,21,22]. Other microRNAs for IAV unique genes are miR-145 for hemagglutinin protein (HA), miR-216b for neuraminidase protein (NA), let-7c for matrix protein 1 (M1) and miR-146a for NS1 [4,10,16,21,22,23]. Unique microRNAs for nucleoprotein (NP) have yet to be discovered; instead, its regulating microRNAs (miR-9, -16, -21, -27a, -29a, -30a, -33a, -136 and -222) have multiple targets [4,6,10,15,24,25]. The miR-92a is another critical miRNA that regulates polymerase basic protein (PB2) and HA [22], whereas the polymerase genes (PB2, PB1 and PA) are regulated by only a few common microRNAs, mainly miR-16, -222 and -4753 [4,21]. Apart from viral gene expression regulations, some host miRNAs also participate in the IAV infection process. Host miRNAs regulate the IAV life cycle by targeting either the viral genome or the innate immune system of the host. IAV pathogenesis regulates some miRNAs to interrupt the antiviral or proviral signaling pathways. Virus-induced suppression or overexpression of certain cellular miRNAs that affect viral pathogenesis is illustrated in Figure 2. For instance, IAV infection reduces miR-548an expression to enhance viral replication [18]. In contrast, IAV infection reduces the expression of miR-4276 [8] to inhibit viral replication. Furthermore, miR-9 expression is increased upon IAV infection to favor virus replication [24], whereas increased miR-146a inhibits viral replication [23]. The regulatory roles of miRNAs in the host–virus interactions remain largely unexplored. A summary of the critical miRNAs in the IAV–host interactions, according to viral pathogenesis, is listed in Table 2.

Viruses also produce some miRNAs. So far, most of the identified viral miRNAs are from DNA viruses. A few hundred miRNAs from RNA viruses have been discovered [33]. Thus far, only one miRNA-like small RNA (miR-HA-3p) has been discovered from an IAV (H5N1). Upon infection, H5N1 generates miR-HA-3p, which suppresses poly(rC)-binding protein 2 (PCBP2) to produce high levels of proinflammatory cytokines and results in high mortality [9]. There have been studies that have attempted to generate an engineered viral-derived miRNA by incorporating pre-miRNA into an IAV genome. One such example was achieved by Varble et al. (2010), where they incorporated pre-miR-124 into the viral genome of the H1N1 IAV to generate functional miR-124 in Madin-Darby Canine Kidney (MDCK) cells without affecting their life cycle [34]. This result suggests that IAVs may use host RNA mechanisms to generate viral miRNAs.

## 3. Influenza A Virus Infection in Relation to Oxidative Stress

The host immune system is affected by oxidative stress, thus, there is an increased susceptibility to viral pathogenesis, including influenza pathogenesis. In contrast, a viral infection produces OS in a host that disrupts its normal homeostasis [35]. The role of oxidants and antioxidants in IAV infection is complicated. Below, we discuss IAV infection and the relationship with OS and host immunity.

**Figure 3:**
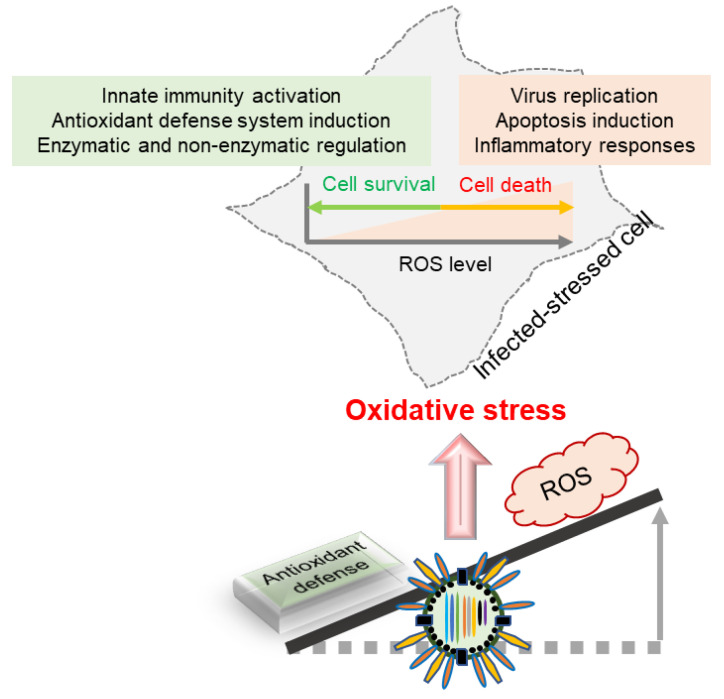
Impact of oxidative stress upon IAV infection. The overproduction of ROS contributes to different pathological changes (i.e., virus replication and cellular inflammation) and leads to apoptosis. On the other hand, medium levels of ROS may induce antioxidant defense systems to control redox homeostasis and promote cell survival in the infected cells.

ROS are critical regulators in many cellular signaling pathways. IAV infection generates ROS that affect host immune systems in many ways, including the activation of monocytes and polymorphonuclear leukocytes, the modulation of host antioxidant responses, and the induction of superoxide dismutase (SOD) [3,36]. As induced SOD is one of the major markers of ROS, there might be a correlation between IAV infection and OS in cells [37]. For instance, it was shown that H3N2 infection in human airway epithelial cells increased Mn-SOD mRNA, which is an important oxidant defense enzyme [38]. Similarly, IAV induces SOD and xanthine oxidase, which, in turn, downregulates the antioxidant concentration, thereby leading to OS [39]. Enhanced OS induces the expression of inflammatory proteins in cells upon IAV infection to mediate cellular immunity. Accordingly, lung inflammation is increased as a consequence of increased inflammatory proteins upon IAV infection [35,40,41]. Viral replication is regulated by antioxidant levels in cells. Hayek et al. (1997) reported that vitamin E (an antioxidant) supplementation is effective in terms of decreasing H3N2 replication in C57BL/6NIA mice [42]. On the other hand, sometimes OS enhances the viral concentrations of influenza viruses [39]. Oxidant–antioxidant imbalance, which regulates ROS levels, is one of the main factors that exacerbates IAV infection and cell damage (Figure 3).

## 4. Interplay among microRNA, Oxidative Stress and IAV Infection

ROS are generated in physiological conditions as a result of regular mitochondrial respiration; however, ROS are also produced in the case of infection and inflammation, leading to pathological conditions. The proper balancing of oxidant and antioxidant factors maintains the homeostasis of the cells. An imbalance between oxidant and antioxidant factors (i.e., OS) regulates several pathophysiological pathways through signal transduction, various transcription factors, and miRNAs. Increasing evidence suggests that miRNAs are involved in ROS production. On the other hand, ROS induce or reduce miRNA expression levels in order to regulate target genes. These differential expressions of miRNAs concerning ROS accumulation subsequently contribute to IAV pathogenesis and/or cellular functions like inflammation or apoptosis. Accordingly, investigating the interactions among miRNA, OS, and IAV infection has become an important endeavor in studying the pathogenesis, treatment, and prevention of IAV infection; however, crosstalk among IAV infection, miRNA and OS has not been discussed elsewhere. This is the first attempt to shed light on this subject. We intend to carry out some analyses or make predictions regarding the interconnection based on reports on IAV–miRNA interaction, miRNA–OS interaction, and OS–IAV interaction regarding the three main events in IAV pathogenesis (i.e., viral replication, cellular inflammation and cell apoptosis).

**Figure 4:**
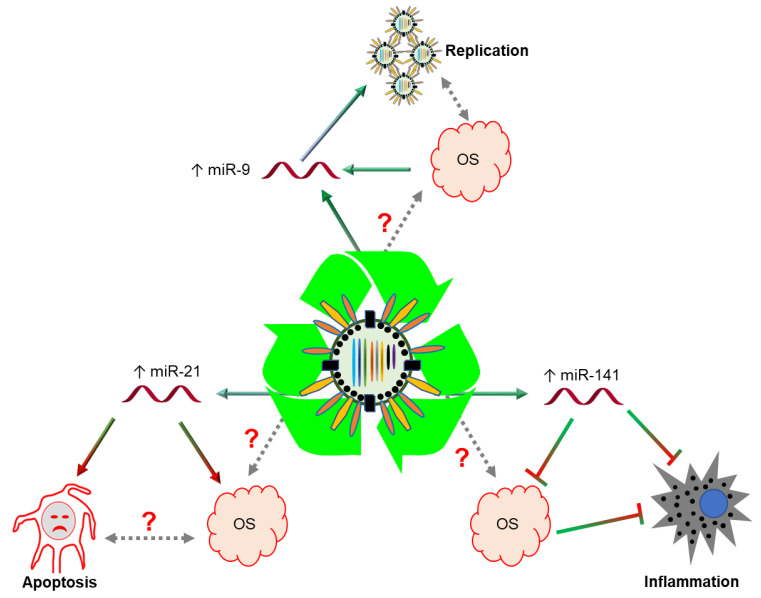
The different interactions among miRNA, OS, and IAV infection. Some specific miRNAs are aberrantly expressed by IAV infection and work as upstream regulators or downstream effectors in OS conditions (→ indicates activation, ⊥ indicates inhibition).

In the case of oxidative stress in different experimental models (cells or animals), differential expressions of miRNAs have been observed (mainly for miR-9, -141, -220a and -21). Interestingly, miR-9 is induced by IAV infection to enhance IAV replication [24], but when OS induces miR-9, it decreases the expression of some mitochondrial target genes, resulting in mitochondrial dysfunction [43]. Thus, it seems that IAV induces miR-9 expression through OS generation, which is the very factor that enhances viral replication and mitochondrial dysfunction. In addition, inflammation is one of the common features of many infectious diseases. Innate immune cells detect infectious agents like IAV, which trigger the inflammatory responses via different cellular factors. Lam et al. (2013) identified the enhanced expression of miR-141 in NCI-H292 cells upon infection with the H1N1 and H5N1 viruses [17]. Cheng et al. (2017) suggested that miR-141 is one of the critical factors that is overexpressed by ROS, followed by nuclear factor erythroid 2-related factor (Nrf2) activation. Nrf2, which is a negative regulator of cellular inflammation, contributes to the anti-inflammatory process by recruiting and regulating antioxidant genes [44]. There is a high chance that the IAV induces ROS to enhance miR-141 expression to decrease cellular inflammation via the Nrf2 pathway. Furthermore, apoptosis is one of the antiviral responses of cells and is operated via programmed cell death when infected cells fail to control IAV replication. Apoptosis could be initiated by a cascade of reactions regulated by different proteins and microRNAs. The critical miRNA in this category is miR-21, which is upregulated by IAV infection to promote apoptosis by regulating the aldehyde dehydrogenase 1 family, member A1 (ALDH1A1) gene [6], although suppressing antioxidant responses in conditions of OS [45]. In contrast, miR-200a, which is another apoptotic regulating miRNA, is downregulated by IAV infection but enhances the antioxidant pathway in ROS conditions [19]. IAV-mediated apoptosis seems to be related to the regulation of the antioxidant pathway by miR-21 and miR-200a in ROS conditions. Overall, these findings suggest that OS represent the upstream regulators or downstream effectors of miRNAs upon IAV infection (Figure 4).

## 5. Clinical Application of miRNAs in IAV Infection and Therapy

As miRNAs have many molecular targets that are connected with IAV infection, they have potential as biomarkers or therapeutics for IAV pathogenesis. A better understanding of the mechanisms underlying miRNA-IAV responses would be helpful to identify novel targets for antiviral agents.

Upon the infection of host cells with an IAV, several selective miRNAs are differentially expressed. Thus, the upregulation or downregulation of miRNAs during IAV infections could be used as biomarkers for infection severity or pathogenesis. While some miRNAs regulate transcription (miR-485 degrades the PB1 transcript [28]), some are involved in translation (miR-9 induces M1 and NP protein expression [24]). Thus, by checking their expression levels, we could distinguish the infection pattern of an IAV; however, some miRNAs are virus sub-type specific, while some target multiple subtypes. For example, miR-3145 is one of the candidates that targets and silences the PB1 gene in multiple subtypes, including H1N1, H3N2, and H5N1 [21]. Additionally, miR-2911 directly targets viral replication by binding with PB2 and NS1 [46]. Furthermore, miRNAs could be a target for antiviral therapy as the downregulation of miR-650 in IAV-infected primary human monocyte-derived dendritic cells (MDDCs) contributes to the establishment of an antiviral state [11]. Lastly, IAV-encoded miR-HA-3p induces proinflammatory cytokines, thus providing a possible efficient treatment strategy to deal with the highly pathogenic infection caused by H5N1 [9]. Taken together, these explanations suggest that miRNAs may directly target IAVs to inhibit their infection and could be a target for the surveillance of IAV outbreaks as therapeutic tools.

## 6. Conclusions

miRNAs have critical roles in many cellular pathways. The dysregulation of miRNAs is intimately linked to viral pathogenesis. Influenza A virus infection causes a serious threat to public health and economies every year. Different IAV strains have different specificities for targeting diverse hosts. IAV–host interactions have been extensively studied; however, they have still not been explored fully. The interaction is governed by a variety of mechanisms that affect the proliferation or elimination of infected cells, where host cell factors play a vital role. miRNA is one of the factors that has been discovered as differentially expressed upon IAV infection. Some of the miRNAs directly regulate host immune responses while some modulate viral replication and gene expression. These miRNA expressions and viral pathogeneses are also linked with ROS generation, which is another critical host factor and concerns maintaining the balance between the oxidants and antioxidants in order to maintain cell homeostasis. ROS regulate several miRNA expressions, and furthermore, miRNA expression also modulates ROS generation upon IAV infection. This interconnecting characteristic between miRNA expression and ROS generation subsequently contributes to IAV pathogenesis. The aforementioned mechanisms show that miRNAs and ROS are key regulators in host–IAV interactions. Several miRNAs have been identified (miR-9, miR-21, miR-141) in relation with ROS, where those miRNAs may be novel targets for biomarkers or therapeutics of IAV pathogenesis. However, several studies are required to validate the crosstalk among miRNA, ROS, and IAV infection to ameliorate or prevent IAV pathogenesis. Finally, it is important to know the interplay among critical host factors like miRNA and ROS in viral pathogenesis in the current pandemic situation, especially in light of the growing fear regarding IAV infections around the globe. In this review, we have attempted to address the host factors that we believe will be important in terms of studying IAV diagnosis and therapeutics.

## Figures and Tables

**Table 1:** List of major up- and down-regulated miRNAs in IAV infection that influence host cells.

Differential Expression	microRNA	Virus Subtypes	Cell/Tissue	Target Gene	Regulatory Function
Upregulation	miR-21 [6]	H5N1	Lung from macaques	*ALDH1A1*	Apoptosis ↑
	miR-223 [6]	H5N1	Lung from macaques	*CYB5A*, *HOXC6*	
	miR-29c [14]	H1N1, H3N2	A549 cells	*BCL2L2*	
	miR-136 [15]	H5N1	A549 cells	*IL-6*	
	let-7a [16]	H1N1	A549 cells	*Cas-3*	Apoptosis ↓
	let-7c [16]	H1N1	A549 cells	*Cas-3*, *EIF2AK2*, *MCM5*, *PA2G4*	
	miR-HA-3p [9]	H5N1	HEK293T	*PCBP2*	Inflammation ↑
	miR-29c [12]	H1N1	A549 cells	*A20*	
	miR-141 [17]	H1N1, H5N1	NCI-H292	*TGF-β2*	Inflammation ↓
	miR-29c [12]	H1N1	A549 cells	*A20*	Antiviral ↓
Downregulation	miR-4276 [8]	H1N1	A549 cells	*COX6C*	Apoptosis ↑
	miR-30 [6]	H5N1	Lung from macaques	*GAL*, *CHI3L1*, *C11orf82*	
	miR-548an [18]	H1N1, H3N2	A549 cells	*NS1ABP*	Apoptosis ↓
	miR-200a [19]	H1N1	Lung from mouse	*IFNAR2*, *STAT4*	Inflammation ↓
	miR-10a [6]	H5N1	Lung from macaques	*BCL6*, *IRAK4*	
	miR-23b [6]	H5N1	Lung from macaques	*CCL2*, *CCL7*, *CSF1*, *IL6R*	
	miR-29c [6]	H5N1	Lung from macaques	*HSPA1A*, *IKBKG*, *NFκB*	
	miR-650 [11]	H1N1	MDDCs	*MxA*	Antiviral ↓

↑ indicates upregulation, ↓ indicates downregulation.

**Table 2:** List of major up- and down-regulated miRNAs in IAV infection during the viral life cycle.

Differential Expression	microRNA	Virus Subtypes	Cell/Tissue	Target Gene	Regulatory Function
Upregulation	miR-9 [24]	H1N1, H3N2	A549 cells	*MCPIP1*	Replication ↑
	miR-34c [26]	H1N1	A549 cells	*PLK4*	
	miR-149-5p [27]	H1N1, H3N2	HEK293T	*M1*, *NP*	
	let-7c [16]	H1N1	MDCK cells	*M1*	Replication ↓
	miR-29c [12]	H1N1	A549 cells	*A20*	
	miR-485 [28]	H5N1	HEK293T	*PB1*	
	miR-146a [23]	H1N1, H3N2	A549 cells	*NF* *κ* *B*	
	miR-101 [29]	H3N2	A549 cells	*NP*	
	miR-203 [30]	H5N1	A549 cells	*DR1*	
	miR-136 [15]	H5N1	MDCK cells	*IL-6*	
Downregulation	miR-124 [31]	H1N1	A549 cells	*ADAMTS7*, *DPP3*, *MST1*	Replication ↑
	miR-548an [18]	H1N1, H3N2	A549 cells	*NS1ABP*	
	miR-124a [31]	H1N1	A549 cells	*CPE*	
	miR-4276 [8]	H1N1	A549 cells	*COX6C*	Replication ↓
	miR-302c [32]	H3N2	A549 cells	*NF* *κ* *B inducing kinase*	

↑ indicates upregulation, ↓ indicates downregulation.

## References

[1] Hangauer M.J., Vaughn I.W., McManus M.T. (2013). Pervasive Transcription of the Human Genome Produces Thousands of Previously Unidentified Long Intergenic Noncoding RNAs. PLoS Genet..

[2] Davis-Dusenbery B.N., Hata A. (2010). Mechanisms of control of microRNA biogenesis. J. Biochem..

[3] Qi X., Zhang H., Wang Q., Wang J. (2016). The NS1 protein of avian influenza virus H9N2 induces oxidative-stress-mediated chicken oviduct epithelial cells apoptosis. J. Gen. Virol..

[4] Makkoch J., Poomipak W., Saengchoowong S., Khongnomnan K., Praianantathavorn K., Jinato T., Poovorawan Y., Payungporn S. (2015). Human microRNAs profiling in response to influenza A viruses (subtypes pH1N1, H3N2, and H5N1). Exp. Biol. Med..

[5] Tambyah P.A., Sepramaniam S., Ali J.M., Chai S.C., Swaminathan P., Armugam A., Jeyaseelan K. (2013). microRNAs in Circulation Are Altered in Response to Influenza A Virus Infection in Humans. PLoS ONE.

[6] Li Y., Li J., Belisle S., Baskin C.R., Tumpey T.M., Katze M.G. (2011). Differential microRNA expression and virulence of avian, 1918 reassortant, and reconstructed 1918 influenza A viruses. Virology.

[7] Moheimani F., Koops J., Williams T., Reid A., Hansbro P.M., Wark P.A.B., Knight D. (2018). Influenza A virus infection dysregulates the expression of microRNA-22 and its targets; CD147 and HDAC4, in epithelium of asthmatics. Respir. Res..

[8] Othumpangat S., Noti J.D., Beezhold D.H. (2014). Lung epithelial cells resist influenza A infection by inducing the expression of cytochrome c oxidase VIc which is modulated by miRNA 4276. Virology.

[9] Li X., Fu Z., Liang H., Wang Y., Qi X., Ding M., Sun X., Zhou Z., Huang Y., Gu H. (2018). H5N1 influenza virus-specific miRNA-like small RNA increases cytokine production and mouse mortality via targeting poly(rC)-binding protein 2. Cell Res..

[10] Nguyen T.H., Liu X., Su Z.Z., Hsu A.C.-Y., Foster P.S., Yang M. (2018). Potential Role of MicroRNAs in the Regulation of Antiviral Responses to Influenza Infection. Front. Immunol..

[11] Pichulik T., Khatamzas E., Liu X., Brain O., Garcia M.D., Leslie A., Danis B., Mayer A., Baban D., Ragoussis J. (2015). Pattern recognition receptor mediated downregulation of microRNA-650 fine-tunes MxA expression in dendritic cells infected with influenza A virus. Eur. J. Immunol..

[12] Zhang X., Dong C., Sun X., Li Z., Zhang M., Guan Z., Duan M. (2014). Induction of the cellular miR-29c by influenza virus inhibits the innate immune response through protection of A20 mRNA. Biochem. Biophys. Res. Commun..

[13] Rosenberger C.M., Podyminogin R.L., Navarro G., Zhao G.-W., Askovich P.S., Weiss M.J., Aderem A. (2012). miR-451 Regulates Dendritic Cell Cytokine Responses to Influenza Infection. J. Immunol..

[14] Guan Z., Shi N., Song Y., Zhang X., Zhang M., Duan M. (2012). Induction of the cellular microRNA-29c by influenza virus contributes to virus-mediated apoptosis through repression of antiapoptotic factors BCL2L2. Biochem. Biophys. Res. Commun..

[15] Zhao L., Zhu J., Zhou H., Zhao Z., Zou Z., Liu X., Lin X., Zhang X., Deng X., Wang R. (2015). Identification of cellular microRNA-136 as a dual regulator of RIG-I-mediated innate immunity that antagonizes H5N1 IAV replication in A549 cells. Sci. Rep..

[16] Ma Y.-J., Yang J., Fan X.-L., Zhao H.-B., Hu W., Li Z.-P., Yu G.-C., Ding X.-R., Wang J.-Z., Bo X.-C. (2012). Cellular microRNA let-7c inhibits M1 protein expression of the H1N1 influenza A virus in infected human lung epithelial cells. J. Cell. Mol. Med..

[17] Lam W.-Y., Yeung A.C.-M., Ngai K.L.-K., Li M.-S., To K.F., Tsui S.K.W., Chan P.K.S. (2013). Effect of avian influenza A H5N1 infection on the expression of microRNA-141 in human respiratory epithelial cells. BMC Microbiol..

[18] Othumpangat S., Noti J.D., Blachere F.M., Beezhold D.H. (2013). Expression of non-structural-1A binding protein in lung epithelial cells is modulated by miRNA-548an on exposure to influenza A virus. Virology.

[19] Li Y., Chan E.Y., Li J., Ni C., Peng X., Rosenzweig E., Tumpey T.M., Katze M.G. (2010). MicroRNA Expression and Virulence in Pandemic Influenza Virus-Infected Mice. J. Virol..

[20] Song L., Liu H., Gao S., Jiang W., Huang W. (2010). Cellular MicroRNAs Inhibit Replication of the H1N1 Influenza A Virus in Infected Cells. J. Virol..

[21] Khongnomnan K., Makkoch J., Poomipak W., Poovorawan Y., Payungporn S. (2015). Human miR-3145 inhibits influenza A viruses replication by targeting and silencing viral PB1 gene. Exp. Biol. Med..

[22] He T., Feng G.-H., Chen H., Wang L., Wang Y. (2009). Identification of host encoded microRNAs interacting with novel swine-origin influenza A (H1N1) virus and swine influenza virus. Bioinformation.

[23] Terrier O., Textoris J., Carron C., Marcel V., Bourdon J.-C., Rosa-Calatrava M. (2013). Host microRNA molecular signatures associated with human H1N1 and H3N2 influenza A viruses reveal an unanticipated antiviral activity for miR-146a. J. Gen. Virol..

[24] Dong C., Sun X., Guan Z., Zhang M., Duan M. (2016). Modulation of influenza A virus replication by microRNA-9 through targeting MCPIP1. J. Med. Virol..

[25] Hu Y., Jiang L., Lai W., Qin Y., Zhang T., Wang S., Ye X. (2016). MicroRNA-33a disturbs influenza A virus replication by targeting ARCN1 and inhibiting viral ribonucleoprotein activity. J. Gen. Virol..

[26] Bakre A., Andersen L.E., Meliopoulos V., Coleman K., Yan X., Brooks P., Crabtree J., Tompkins S.M., Tripp R.A. (2013). Identification of Host Kinase Genes Required for Influenza Virus Replication and the Regulatory Role of MicroRNAs. PLoS ONE.

[27] Peng S., Wang J., Wei S., Li C., Zhou K., Hu J., Ye X., Yan J., Liu W., Gao G.F. (2018). Endogenous Cellular MicroRNAs Mediate Antiviral Defense against Influenza A Virus. Mol. Ther. Nucleic Acids.

[28] Ingle H., Kumar S., Raut A.A., Mishra A., Kulkarni D.D., Kameyama T., Takaoka A., Akira S., Kumar H. (2015). The microRNA miR-485 targets host and influenza virus transcripts to regulate antiviral immunity and restrict viral replication. Sci. Signal..

[29] Sharma S., Chatterjee A., Kumar P., Lal S.K., Kondabagil K. (2020). Upregulation of miR-101 during Influenza A Virus Infection Abrogates Viral Life Cycle by Targeting mTOR Pathway. Viruses.

[30] Zhang S., Li J., Li J., Yang Y., Kang X., Li Y., Wu X., Zhu Q., Zhou Y., Hu Y. (2018). Up-regulation of microRNA-203 in influenza A virus infection inhibits viral replication by targeting DR1. Sci. Rep..

[31] Meliopoulos V.A., Andersen L.E., Brooks P., Yan X., Bakre A., Coleman J.K., Tompkins S.M., Tripp R.A. (2012). MicroRNA Regulation of Human Protease Genes Essential for Influenza Virus Replication. PLoS ONE.

[32] Gui S., Chen X., Zhang M., Zhao F., Wan Y., Wang L., Xu G., Zhou L., Yue X., Zhu Y. (2015). Mir-302c mediates influenza A virus-induced IFNβ expression by targeting NF-κB inducing kinase. FEBS Lett..

[33] Qi P., Han J.X., Lu Y., Wang C., Bu F.F. (2006). Virus-encoded microRNAs: Future therapeutic targets?. Cell. Mol. Immunol..

[34] Varble A., Chua M.A., Perez J.T., Manicassamy B., García-Sastre A., Tenoever B.R. (2010). Engineered RNA viral synthesis of microRNAs. Proc. Natl. Acad. Sci. USA.

[35] Liu M., Chen F., Liu T., Chen F., Liu S., Yang J. (2017). The role of oxidative stress in influenza virus infection. Microbes Infect..

[36] Peterhans E., Grob M., Burge T., Zanoni R. (1987). Virus-Induced Formation of Reactive Oxygen Intermediates in Phagocytic Cells. Free. Radic. Res. Commun..

[37] Hong S.C., Murale D.P., Jang S.-Y., Haque M., Seo M., Lee S., Woo D.H., Kwon J., Song C.-S., Kim Y.K. (2018). Discrimination of Avian Influenza Virus Subtypes using Host-Cell Infection Fingerprinting by a Sulfinate-based Fluorescence Superoxide Probe. Angew. Chem. Int. Ed..

[38] Jacoby D.B., Choi A.M. (1994). Influenza virus induces expression of antioxidant genes in human epithelial cells. Free. Radic. Biol. Med..

[39] Peterhans E. (1997). Oxidants and Antioxidants in Viral Diseases: Disease Mechanisms and Metabolic Regulation. J. Nutr..

[40] Shi M.M., Godleski J.J., Paulauskis J.D. (1996). Regulation of Macrophage Inflammatory Protein-1 mRNA by Oxidative Stress. J. Biol. Chem..

[41] Selemidis S., Seow H.J., Broughton B.R.S., Vinh A., Bozinovski S., Sobey C.G., Drummond G.R., Vlahos R. (2013). Nox1 Oxidase Suppresses Influenza A Virus-Induced Lung Inflammation and Oxidative Stress. PLoS ONE.

[42] Hayek M.G., Taylor S.F., Bender B.S., Han S.N., Meydani M., Smith D.E., Eghtesada S., Meydani S.N. (1997). Vitamin E Supplementation Decreases Lung Virus Titers in Mice Infected with Influenza. J. Infect. Dis..

[43] Meseguer S., Martínez-Zamora A., Garcia-Arumi E., Andreu A.L., Armengod M.-E. (2014). The ROS-sensitive microRNA-9/9* controls the expression of mitochondrial tRNA-modifying enzymes and is involved in the molecular mechanism of MELAS syndrome. Hum. Mol. Genet..

[44] Cheng L.-B., Li K.-R., Yi N., Li X.-M., Wang F., Xue B., Pan Y.-S., Yao J., Jiang Q., Wu Z.-F. (2017). miRNA-141 attenuates UV-induced oxidative stress via activating Keap1-Nrf2 signaling in human retinal pigment epithelium cells and retinal ganglion cells. Oncotarget.

[45] La Sala L., Mrakic-Sposta S., Micheloni S., Prattichizzo F., Ceriello A. (2018). Glucose-sensing microRNA-21 disrupts ROS homeostasis and impairs antioxidant responses in cellular glucose variability. Cardiovasc. Diabetol..

[46] Zhou Z., Li X., Liu J., Dongxia H., Chen Q., Liu J., Kong H., Zhang Q., Qianyi Z., Hou D. (2015). Honeysuckle-encoded atypical microRNA2911 directly targets influenza A viruses. Cell Res..

